# “Not just another walking program”: Everyday Activity Supports You (EASY) model—a randomized pilot study for a parallel randomized controlled trial

**DOI:** 10.1186/2055-5784-1-4

**Published:** 2015-01-12

**Authors:** Maureen C Ashe, Meghan Winters, Christiane A Hoppmann, Martin G Dawes, Paul A Gardiner, Lora M Giangregorio, Kenneth M Madden, Megan M McAllister, Gillian Wong, Joseph H Puyat, Joel Singer, Joanie Sims-Gould, Heather A McKay

**Affiliations:** 1 Centre for Hip Health and Mobility, 7F-2635 Laurel Street, Vancouver, BC V5Z 1 M9 Canada; 2 Department of Family Practice, University of British Columbia (UBC), 320-5950 University Boulevard, Vancouver, BC V6T 1Z3 Canada; 3 Faculty of Health Sciences, Simon Fraser University, Blusson Hall Rm 11522, 8888 University Drive, Burnaby, BC V5A 1S6 Canada; 4 UBC Department of Psychology, 2136 West Mall, Vancouver, BC V6T 1Z4 Canada; 5 School of Public Health, The University of Queensland, Level 3, Public Health Building, Herston Rd, Herston, Queensland 4006 Australia; 6 Translating Research Into Practice (TRIP) Centre, Mater Research Institute-The University of Queensland, Level 3 Aubigny Place, Raymond Terrace, South Brisbane, Queensland 4101 Australia; 7 Department of Kinesiology, University of Waterloo, 200 University Ave W, Waterloo, Ontario N1H 8 K4 Canada; 8 UBC Department of Medicine, Division of Geriatric Medicine, Room 7185, 2775 Laurel Street, Vancouver, BC N2L 3G1 Canada; 9 UBC School of Population and Public Health, 2206 East Mall, Vancouver, BC V6T 1Z3 Canada; 10 Centre for Health Evaluation and Outcomes Sciences, St. Paul’s Hospital, 588-1081 Burrard Street, Vancouver, BC V6Z 1Y6 Canada; 11 UBC Department of Orthopaedics, 3114-910 West 10th Avenue, Vancouver, BC V5Z 1 M9 Canada

**Keywords:** Sedentary lifestyle, Motor activity, Self-management, Retirement, Women

## Abstract

**Background:**

Maintaining physical activity is an important goal with positive health benefits, yet many people spend most of their day sitting. Our Everyday Activity Supports You (EASY) model aims to encourage movement through daily activities and utilitarian walking. The primary objective of this phase was to test study feasibility (recruitment and retention rates) for the EASY model.

**Methods:**

This 6-month study took place in Vancouver, Canada, from May to December 2013, with data analyses in February 2014. Participants were healthy, inactive, community-dwelling women aged 55–70 years. We recruited through advertisements in local community newspapers and randomized participants using a remote web service. The model included the following: group-based education and social support, individualized physical activity prescription (called Activity 4-1-1), and use of a Fitbit activity monitor. The control group received health-related information only. The main outcome measures were descriptions of study feasibility (recruitment and retention rates). We also collected information on activity patterns (ActiGraph GT3X+ accelerometers) and health-related outcomes such as body composition (height and weight using standard techniques), blood pressure (automatic blood pressure monitor), and psychosocial variables (questionnaires).

**Results:**

We advertised in local community newspapers to recruit participants. Over 3 weeks, 82 participants telephoned; following screening, 68% (56/82) met the inclusion criteria and 45% (25/56) were randomized by remote web-based allocation. This included 13 participants in the intervention group and 12 participants in the control group (education). At 6 months, 12/13 (92%) intervention and 8/12 (67%) control participants completed the final assessment. Controlling for baseline values, the intervention group had an average of 2,080 [95% confidence intervals (CIs) 704, 4,918] more steps/day at 6 months compared with the control group. There was an average between group difference in weight loss of −4.3 [95% CI −6.22, −2.40] kg and reduction in diastolic blood pressure of −8.54 [95% CI −16.89, −0.198] mmHg, in favor of EASY.

**Conclusions:**

The EASY pilot study was feasible to deliver; there was an increase in physical activity and reduction in weight and blood pressure for intervention participants at 6 months.

**Trial registration:**

ClinicalTrials.gov identifier: NCT01842061

**Electronic supplementary material:**

The online version of this article (doi:10.1186/2055-5784-1-4) contains supplementary material, which is available to authorized users.

## Background

The World Health Organization ranked physical inactivity as the world’s fourth most important risk factor for mortality [1], in part, because we engineered activity out of our everyday lives [2]. While there is abundant evidence that physical activity prevents chronic disease [3], many older adults fail to meet guideline recommendations [4–6]. Even those who meet guideline physical activity recommendations spend most of the day sitting [7, 8] such that sedentariness is emerging as an independent risk factor for morbidity [9] and mortality [10].

Women, in particular, are affected by higher rates of sedentary behavior [11] which places them at increased risk of developing some chronic diseases [12]. In Canada, about half of women aged 45–64 years engage in leisure-time physical activity, lower than the levels of men for the same age range [12]. Further, among women aged 60–69 years, 65% had a waist circumference considered high risk and a third of these women were obese [13]. When their physical activity was measured objectively (using accelerometry), they had only 12 min/day of moderate to vigorous physical activity (MVPA) and spent 10 h of the waking day sitting [13]. Fewer than 5% of these women received “excellent” or “very good” for their fitness test. Given the physical inactivity, sedentary behavior, obesity, and the challenge of maintaining healthy behaviors, it is of little surprise that compared with men, women are at an elevated risk for developing some chronic diseases and live with more disability in later life [14–16]. Previous studies with older women highlighted that timing is a key factor for the uptake of healthy behaviors [17], and retirement may be an opportune time to focus on their own health to reduce the risk for chronic disease in later life [18].

In their systematic review, Hobbs and colleagues [19] highlight successful models for physical activity, yet they concluded that these results do not extend beyond *1 year*. Further, they do not discuss response to these interventions separately for men and women. Ross and colleagues [20] demonstrated a significant reduction in body size for middle-aged Canadian men and women who adopted a physician-initiated behavioral change intervention. However, results in women were not sustained at 1-year follow-up [20]. Further, in a Diabetes Prevention Program study, men were more likely to meet physical activity goals compared with women [21].

Our goal is to develop *sustainable* physical activity models that encourage people to be more active in ways that are integrated into their lifestyle [22]. We hypothesize that simple strategies, such as including more activities of daily living (e.g., household tasks, gardening) and/or daily public transit use [23], encourage movement with the downstream benefits of more physical activity such as increased fitness and enhanced social engagement and quality of life [24]. These more routine ways to create positive physical activity habits [22] may, in the long run, be easier to maintain in daily life. We further hypothesized that an intervention based on everyday activities, delivered in a group setting, may be positive for women at retirement. Thus, studies are needed to evaluate the potential for inactive middle-age women to become physically active as a means to socialize and enhance health [25].

Despite elevated attention paid to (and risk for) increased sitting time in middle-aged and older adults, there are relatively few sedentary behavior interventions that specifically target this age group. Gardiner and colleagues tested feasibility of a brief goal-setting strategy to reduce sitting time in adults 60+ years [26] and noted a 3.2% reduction in sitting over 2 weeks. Fitzsimons and colleagues also tested the feasibility of a brief individualized intervention that resulted in a 24 min/day reduction in sitting [27]. Prince and colleagues [28], in their systematic review of interventions (with a physical activity and/or sedentary behavior focus) to reduce sitting time in adults, noted that only two physical activity studies that targeted older adults had a positive effect on sedentary behavior [29, 30]. Further, of the sedentary behavior studies and/or sedentary behavior + physical activity studies, only one study specifically focused on older adults [31]. They used a quasi-experimental 8-week multi-prong intervention and achieved a significant reduction in sitting time [31].

The Everyday Activity Supports You (EASY) model, grounded in the social-ecological model [32] and guided by the social cognitive theory [33], aims to encourage sustainable adoption of more activity, by first reducing sitting time and then incrementally increasing physical activity. The success of simple strategies (such as beginning by sitting less) has the potential to support self-efficacy (mastery) [33, 34] and provides a foundation to gradually add more daily physical activity—a “sit less to move more” approach. The novelty of this approach is that it acknowledges the physiological distinction of sedentary behavior (too much sitting) [7] and physical inactivity [35], *but* seeks to utilize behavior change techniques (BCTs), such as graded tasks (small incremental changes in daily routine) [36], for long-term habit formation. Thus, we hypothesized that the process of increasing physical activity begins with *sitting less*.

The EASY model aims to extend previous work and specifically targets reduced sitting time, to initially increase physical activity; to our knowledge, this approach has not been studied in women at retirement age. Our primary objective for this phase was to test study feasibility by measuring participant recruitment and retention rates. Second, we sought information on participants’ satisfaction with the program. Finally, we aimed to determine the timing and resource requirements for program delivery and outcome assessments. The secondary health objectives were to determine the effect of the model on physical activity, sedentary behavior, and health-related outcomes. Knowledge of this information sets the foundation for future effectiveness studies and scale up and spread of the intervention.

## Methods

The social ecological model [32] identifies the impact of multiple levels of societal influence that span individual to policy and their influence on health outcomes, health promotion, and behavior change. In this study, we aimed to develop a program that addressed the intersection of macroscale and microscale “levels” that alone and together influence the health of individuals and populations, with particular regard to person, people (social environment), and places (community/built environment). The values of participatory action research [37], especially components that relate to social learning, collective problem solving, capacity building, and empowerment (self-efficacy), very nicely complement the elements of the social ecological model. We included in the EASY model a participatory focus that generated participant collaboration and reflexivity known to optimize the accessibility, uptake, effectiveness, and, in future, scale up of effective, sustainable programs to the wider community [38].

### Trial design and setting

This was a phase II [39] parallel 1:1 RCT pilot of the EASY model lifestyle program (ClinicalTrials.gov identifier: NCT01842061). The intervention was 6 months in duration, and there were three data collection periods: baseline (May 2013), midpoint (August 2013), and final assessment (December 2013); data were analyzed in February 2014. The study took place in Vancouver, British Columbia. Metro Vancouver (census metropolitan area) has a population of approximately 2.3 million residents [40], with 13.5% of the population 65+ years [41]. We worked with a local community centre, within Vancouver, and originally targeted our recruitment strategies to that neighborhood. However, the local newspaper was accessible to residents from all regions of Vancouver.

### Participants

We included healthy community-dwelling women 55–70 years of age who self-identified as not engaging (in the previous 3 months) in strength training or more than 30 min of brisk walking or moderate exercise/week [42, 43] and were able to climb a flight of stairs and walk 400 m [44]. We excluded participants who received treatment for any medical conditions that precluded walking regularly. This study was approved by the University of British Columbia and the Vancouver Coastal Health Research Institute IRB. All participants provided the following: (1) written informed consent to participate, and (2) if they answered yes to any question to the Physical Activity Readiness Questionnaire Plus (PAR-Q+ [45]) during in-person screening by the exercise physiologist, they were required to obtain written permission from their family physician to participate.

### Recruitment

In May 2013, we placed advertisements in four community newspapers and sent emails to relevant groups (e.g., health professional associations) and placed posters in the local neighborhood library and community center.

### Baseline assessment

We completed all baseline assessments over a 9-day period from May 21–30, 2013. Participants underwent a 60–75-min in-person assessment (including the screen by the exercise physiologist) and took home a package containing questionnaires and an accelerometer (ActiGraph GT3X+, ActiGraph LLC, Pensacola, FL).

### Randomization

An independent statistical consultant set up the web-based randomization process to assign eligible participants to intervention or control groups by remote allocation, using permuted blocks of sizes 2 and 4. No one directly involved in the project had access to allocation codes. As this was a pilot study, no stratification was used. After all baseline assessments were completed, the study coordinator entered participant identification numbers into the web service to allocate groups. All interactions with the web service were automatically logged and included information on study identification number, group allocation, and randomization date/time.

#### Protection against bias

Treatment allocation was concealed as described above. Only those who did not deliver the intervention (measurement team) were blinded to group allocation.

### Intervention group

**Figure 1:**
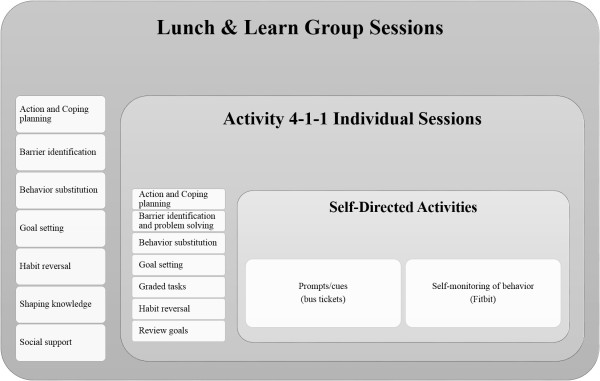
**An overview of the behavior change techniques (BCTs).** The BCTs were based on the Taxonomy by Abraham and Michie [36, 46], and underpins the three main components of the Everyday Activity Supports You (EASY) model.

The EASY model is focused on reduced sitting time to encourage more physical activity. It has three main elements: group-based education and social support, individualized physical activity prescription (which we named Activity 4-1-1), and use of an activity monitor [Fitbit One (Fitbit Inc., San Francisco, CA)]. The Fitbit is an accelerometer that provides immediate feedback on activities including daily step counts, distance walked, and stairs climbed. It also provides an online tool to monitor other behaviors (sleep and nutrition) and facilitate social networking and/or friendly competitions. Participants were not required to share Fitbit data with the research team. However, the exercise professionals (personal trainer or exercise physiologist) used recorded step counts (from the Fitbit) to calculate step increases at individualized sessions. Figure 1 and Additional file 1: Table S1 provide a summary of the BCTs utilized in the EASY model. Additional file 1: Figures S1 and S2 are examples of handouts used and generated during the group-based sessions.

There were two main phases: (1) a *ramp up* (consisting of four weekly sessions) and (2) an *activation* phase (consisting of five monthly sessions). During both phases, the first hour of each session was a participatory component on selected physical activity-related topics. In the second hour of each session, there were Activity 4-1-1 sessions and concurrent brainstorming group sessions. The Activity 4-1-1 was the individual time that study participants had with an exercise professional to discuss their progress to date, goals, and individual walking (step count) prescription. We called the individual time spent with the exercise professions as Activity 4-1-1 because it was the opportunity that study participants could seek activity “information” from the exercise professionals. During these sessions, participants had a 10–15-min individual meeting with the exercise professional for a physical activity prescription. Concurrently, the study coordinator ran group discussions with the remaining participants on topics such as dealing with setbacks, barriers to being active, etc. The information generated from the group discussions was typed and provided to the participants the following week (please see Additional file 1: Figures S1 and S2 and Table S1). At two time points over the 6 months (months 1 and 4), intervention participants were given a booklet of 10 transit tickets to encourage use of public transportation.

1. Ramp up: The goals of this phase were to become familiar with the Fitbit, set activity goals, and develop strategies to reduce sedentary behavior. The study coordinator led the sessions with support from two exercise professionals who were in attendance at every session. This phase focused on reminding participants to reduce their sitting time and then gradually begin introducing more activity into daily routines. In addition, the following information was provided: practical information on common stretches and opportunities to practice them while the personal trainer checked posture and a session from a pedorthist on choosing appropriate footwear for physical activity.

In the Activity 4-1-1 (individual) sessions, participants were encouraged (if able) to increase their step counts by 5% at each visit, based initially on their first week’s step counts from the research accelerometer data and in subsequent sessions based on their Fitbit step counts. Participants were not asked to aim for a target daily step count (e.g., 10,000 steps/day). Rather, it was discussed that there were common targets (e.g., 7,000 steps/day [47]), but highlighted that each person responds differently to increasing physical activity.

2. Activation: For this phase, one monthly class was offered to the intervention group. The education topics included the following: (1) how to take public transportation; (2) the importance of exercise; (3) bone health and falls prevention; (4) a dietician-guided tour of a grocery store; (5) gearing up for physical activity tips, tricks, and safety; and (6) the final session on how to sustain activity patterns. Our aim for the grocery store tour was twofold to provide an opportunity for community-engagement to encourage physical activity to local destinations and provide a general overview of a grocery store layout (with an emphasis on nutrition-rich food).

### Control group

We provided control participants with separate monthly education sessions. Three education topics were similar to the intervention group (how to take public transportation, bone health and falls prevention, and personal safety), but they did *not* receive information on the importance of exercise or how to sustain an active lifestyle. Control group participants had no interactions with the exercise professionals nor did they receive Fitbit monitors. Control participants received a $20 gift certificate at two time points (consistent with the delivery of the transit tickets to the intervention group). Based on feedback from control participants, the EASY model activity components (Fitbit and Activity 4-1-1) were offered to this group at the end of the study (after the final assessment).

### Primary outcome measures

The main objective of this pilot study was to determine recruitment and retention rates. Second, we sought information on participants’ satisfaction with the program. Finally, we aimed to determine appropriateness of program delivery, resources required, and the specific components of the outcomes assessment to evaluate the EASY model in a larger trial. We defined success for recruitment for this feasibility study as enrolling up to 15 but no less than eight participants/group within the short timeframe (3 weeks); this permitted running two parallel arms of the study. We acknowledge that enrolling 15 participants/group would not constitute successful recruitment in a larger trial. However, these targets aim to provide us an estimate of interest in our program. We defined success for participant retention as 80% of study participants completing the final assessment. We also asked participants to rate their satisfaction with the program (scored out of a possible 10 points, where 1 was not satisfied and 10 was highly satisfied).

### Health outcome measures

As a secondary aim, we collected health outcome measures at three times during the study to determine feasibility of our assessment protocol and a preliminary estimate of treatment effect.

### Activity patterns

We assessed physical activity (average daily step count) using ActiGraph GTX3+ (LLC, Fort Walton Beach, FL, USA) tri-axial accelerometers worn at the hip during waking hours for 7 days at each time point; Fitbits were only for participants’ daily use.

We collected the following outcomes: MVPA (min/day) and sedentary behavior (min/day and percentage/day). We defined sedentary behavior as “any waking behavior characterized by an energy expenditure ≤1.5 METs while in a sitting or reclining posture” [35]. We standardized it by reporting percentage of sedentary time or by including both sedentary time (min) and total wear time (min) in the models.

#### Accelerometry assumptions and data handling

We reintegrated raw data (collected at 30 Hz) to 60-s epochs; we considered more than 90 min of continuous zeroes as non-wear time. To be included in the analysis, accelerometry data had to include three or more valid days (8 h/day) of wear time. Based on our systematic review [48], we used the following cut points: ≤99 counts/min as sedentary time [49], 100–1,951 counts/min as light physical activity, and ≥1,952 counts/min for MVPA [50]. We used ActiLife (Version 6.10.0) to clean and analyze accelerometry data.

### Social connectedness, self-rated health, self-efficacy, and intentions for physical activity

We used the modified Medical Outcomes Study Social Support Survey Instrument [51] to assess the effect of the intervention on participants’ social network. We assessed self-rated health with the visual analogue scale (out of 100 points) [52]. We recorded participants’ beliefs around physical activity with the McAuley Exercise Self-efficacy Scale [53], and we used the Behavioral Intentions for Physical Activity questionnaire [54] to assess participants’ physical activity goals.

### Body composition/blood pressure

We measured height (cm) and weight (kg; Conair Corporation, Glendale, AZ) using standard methods (average of two measurements) and calculated BMI as weight (kg)/height (m)^2^. We used BpTRU BPM-200 (BpTRU Medical Devices, Coquitlam, BC) automatic cuff was used to obtain blood pressure (mmHg) in study participants at baseline and final assessments (average of two trials).

### Descriptive information

We collected the following data: year of birth, highest level of education, and the Functional Comorbidity Index [55].

### Adverse events monitoring

We requested that study participants notify the study coordinator of any adverse events throughout the course of the study. All adverse events were recorded by the study coordinator, and if deemed a serious adverse event (SAE), a physician not involved in the study was appointed to review them.

### Statistical analysis

We did not conduct a formal sample size calculation for this feasibility study; rather, we aimed to recruit sufficient participants to generate estimates of variability for our outcome measures and to generate a preliminary estimate of effect for the intervention.

We described participant characteristics using mean and standard deviations or medians and interquartile range if appropriate. *To address feasibility*, we calculated recruitment and retention rates and report percentage; we also report participants’ program satisfaction as mean (standard deviation). *For the health outcome variables*, there were two sets of analyses. First, we estimated average change by fitting separate linear regression models for each of the health outcome variables using group allocation as the only independent variable. We performed a second set of analyses, with constructed models that included baseline values as covariates [56]. We report the regression coefficients and *P* values for the group allocation variable and *R* ^2^ values from the regression analyses to provide an estimate of model fit. Due to skewed residuals, we used log-(Y)-transformed values for all significance tests and regression analyses for physical activity (step counts and MVPA). The *P* values and model *R* ^2^ values for MVPA and step counts were obtained from analyses of log-transformed data. The beta coefficients, however, were obtained from analyses of original data to allow for interpretation of treatment effects as the arithmetic mean of the differences in MVPA and step counts between the two study groups. Further, we estimated confidence intervals (CIs) and standard errors of intervention effects for these variables (step counts and MVPA) through nonparametric bootstrapping using 1,000 resamples with random seed set to a value of 1,234. We used Stata version 12 (StataCorp, College Station, TX, USA).

## Results

This study ran from May to December 2013 inclusive of recruitment and final assessment. We were able to utilize all outcome measurement instruments initially proposed, including the request for study participants to wear an accelerometer for 7 days following assessment.

**Figure 2:**
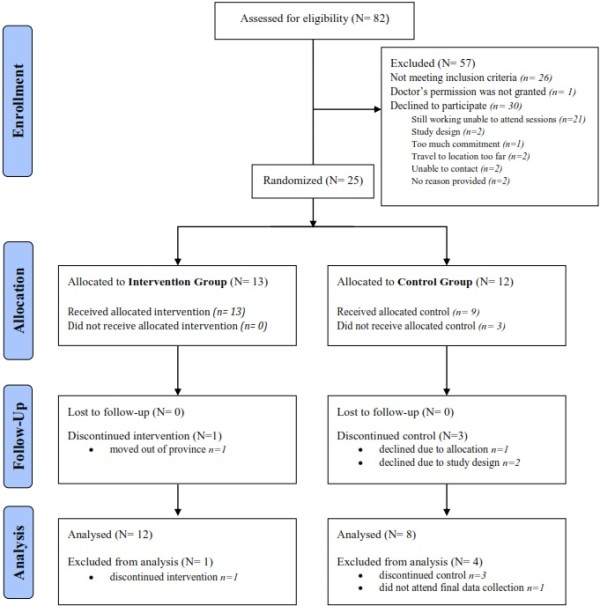
**CONSORT 2010 Flow diagram for the EASY-Pilot study.**

Eighty two participants responded to newspaper ads, 56 participants were eligible following telephone screen by a trained research assistant and 26 participants agreed to take part in the study. Of the study participants who were not eligible to enroll in the study, the three reasons given were: as follows already engaging in an exercise program (*N* = 19), <55 years old (*N* = 5), and >70 years old (*N* = 2). Of 56 eligible participants, 30 declined to enroll. Work demands were cited as the main reason for being unavailable to attend sessions. One participant, who was referred back to her family physician following screening, due to an existing health condition, did not receive physician approval to take part in the study. See Figure 2 for the CONSORT 2010 flow diagram.

Participants had mean (SD) age of 64.1 (4.6) years and median (IQR) of 2 (3) comorbidities. All participants completed secondary school, and some had further education (Table 1). Two intervention participants (2/12, 17%) and four control participants were working (4/8, 50%).

Following baseline assessment, 20 participants were required to return to their family physician to obtain written permission. There were 25/56 (45%) participants who were randomized by remote web service resulting in 13 participants in the intervention group and 12 participants in the control group. At 6 months, 20/25 (80%) of study participants completed the final assessment, including 12/13 (92%) intervention and 8/12 (67%) control participants (Figure 2). During the course of the study, one intervention and one control participant each sustained an SAE that were deemed unrelated to study participation; however, the control participant’s injury prevented her from wearing the accelerometer at the final assessment.

**Table 1:** **Characteristics of study participants across the three time points of the study**

	Baseline		Midpoint, 3 months		Final, 6 months	
	Control	Intervention	Control	Intervention	Control	Intervention
	***N***= 8	***N***= 12	***N***= 8	***N***= 12	***N***= 8	***N***= 12
Age (years) mean (SD)	63.1 (4.8)	64.8 (4.6)				
BMI	32.9 (6.8)	26.9 (6.8)				
Education: secondary education+	8	12				
Employment: working	4	2				
Weight (kg)						
Mean (SD)	90.19 (18.94)	69.69 (19.32)	90.06 (18.50)	67.93 (18.55)	91.6 (19.04)	67.33 (18.57)
Systolic blood pressure (mmHg)						
Mean (SD)	139.94 (13.33)	127.92 (16.31)	–	–	138.56 (14.55)	117.04 (18.73)
Diastolic blood pressure (mmHg)						
Mean (SD)	83.44 (9.31)	77.29 (6.85)	–	–	83.38 (8.93)	70.88 (9.57)
Behavior intentions/5						
Median (IQR)	3.6 (1.1)	4.4 (1.5)	3.3 (1.3)	4.0 (1.3)	3.1 (1.3)	4.0 (0.70)
Exercise self-efficacy/10						
Median (IQR)	7.08 (1.75)	7.75 (2.17)	5.17 (1.92)	8.08 (1.92)	6.83 (3.33)	8.25 (1.5)
Self-rated health/100						
Mean (SD)	86.63 (17.52)	79.42 (14.23)	86.5 (14.0)	79.83 (19.19)	77.5 (22.68)	83.75 (16.50)
Social support/100						
Mean (SD)	86.11 (8.42)	79.04 (16.24)	86.45 (8.99)	78.56 (13.82)	88.13 (5.60)	79.39 (11.80)

BMI: body mass index; IQR: interquartile range; SD: standard deviation.

For intervention group sessions, attendance ranged from *n* = 6 (46%) to *n* = 13 (100%); median (IQR) was 10 (3.8) participants/session. Control sessions had lower attendance at education sessions [median (IQR) 6.5 (1.8) participants]. Overall, at 6 months, participants in the intervention group rated their satisfaction with the program as [mean (SD)] 9 (1) points; it was 9 (1) points for control group participants.

We provide initial estimates of treatment effect for the EASY model; however, these early results may not be replicated in a larger, definitive trial. At the 3- and 6-month time point intervention, participants increased their daily step counts and MVPA and decreased sedentary time; there was a larger increase at midpoint (August) compared with final assessment (November). Control participants decreased both step counts and MVPA over the 6-month time period; they also increased sitting time. There was a statistically significant between-group difference for step count 2,080 [95% CI 704, 4,918] at the final assessment in both the unadjusted (*P* = 0.046) and adjusted models (*P* = 0.040) favoring EASY. There were no statistically significant differences between groups for MVPA or sedentary time (Tables 2 and 3).

**Table 2:** **Physical activity and sedentary behavior outcomes for the study groups at three time points**

	Baseline		Midpoint, 3 months		Final, 6 months	
	Control	Intervention	Control	Intervention	Control	Intervention
	(***N***= 7)	(***N***= 12)	(***N***= 7)	(***N***= 12)	(***N***= 7)	(***N***= 12)
Step count (steps/day)						
Mean (SD)	5,340 (1,966)	6,402 (2,534)	4,251 (1,185)	8,038 (3,317)	4,593 (663)	7,606 (3,917)
Median (IQR)	4,786 (4,313)	5,918 (4,599)	4,002 (1,925)	6,914 (5,936)	4,444 (988)	6,295 (5,255)
MVPA (min/day)						
Mean (SD)	24.33 (32.69)	23.39 (15.21)	12.22 (9.50)	34.31 (24.32)	13.49 (8.03)	33.06 (28.83)
Median (IQR)	11.33 (27.57)	19.07 (24.5)	7.43 (13.57)	34.36 (28.79)	10.00 (18.00)	27.36 (34.36)
Sedentary behavior (%)						
Mean (SD)	62.42 (12.95)	67.75 (7.45)	70.66 (4.95)	65.56 (7.20)	67.23 (4.68)	66.17 (7.06)

MVPA: moderate to vigorous physical activity.

**Table 3:** **Average difference between groups for physical activity measures at final assessment**

	Unadjusted	Adjusted for baseline
Step counts (steps/day)		
*P* value^a^	0.046	0.040
Model *R* ^2a^	0.214	0.678
β coefficient	3,012	2,080
[95% CI]^b^	[1,257, 5,743]	[704, 4,918]
MVPA (min/day)		
*P* value^a^	0.088	0.150
Model *R* ^2a^	0.162	0.555
β coefficient	19.57	19.96
[95% CI] ^b^	[6.18, 41.43]	[5.67, 62.00]
Sedentary behavior (min/day)		
*P* value	0.673	0.489
Model *R* ^2^	0.595	0.665
β coefficient	−12.41	−21.14
[95% CI]	[−73.68, 48.85]	[−84.89, 42.60]

MVPA: moderate to vigorous physical activity; ^a^ *P* values and model *R* ^2^ for MVPA and step counts were obtained from log-transformed data; ^b^95% CIs were estimated from original data using nonparametric bootstrapping with 1,000 resamples and random seed set to 1,234; all other values were obtained from original data.

Table 4 presents results for the between-group analyses of health-related outcomes. There was an average between-group difference of −4.3 [95% CI −6.22, −2.40] kg and reduction in diastolic blood pressure of −8.54 [95% CI −16.89, −0.198] mmHg, in favor of EASY. There were no significant differences between groups for any other variables except unadjusted behavioral intentions, where there was a 0.82 [0.07, 1.56] difference favoring EASY.

**Table 4:** **Average difference between groups at final assessment for health outcomes and other self-reported measures**

	Unadjusted	Adjusted for baseline
Weight (kg)		
*P* value	0.011	<0.001
Model *R* ^2^	0.309	0.994
β coefficient [95% CI]	−24.28 [−42.23, −6.29]	−4.31 [−6.22, −2.40]
Systolic blood pressure (mmHg)		
*P* value	0.014	0.069
Model *R* ^2^	0.294	0.474
β coefficient [95% CI]	−21.52 [−38.04, −5.00]	−14.64 [−30.55, 1.26]
Diastolic blood pressure (mmHg)		
*P* value	0.009	0.045
Model *R* ^2^	0.324	0.525
β coefficient [95% CI]	−12.5 [−21.44,-3.56]	−8.54 [−16.89, −0.198]
Behavioral intentions for physical activity		
*P* value	0.033	0.118
Model *R* ^2^	0.228	0.577
β coefficient [95% CI]	0.817 [0.07, 1.56]	0.469 [−0.13, 1.07]
Exercise self-efficacy		
*P* value	0.128	0.167
Model *R* ^2^	0.124	0.215
β coefficient [95% CI]	1.21 [−0.379,2.80]	1.07 [−0.49,2.64]
Self-rated health		
*P* value	0.484	0.069
Model *R* ^2^	0.028	0.523
β coefficient [95% CI]	6.25 [−12.10, 24.60]	12.56 [−1.10, 26.22]
Social support		
*P* value	0.068	0.150
Model *R* ^2^	0.173	0.564
β coefficient [95% CI]	−8.74 [−18.20, 0.718]	−5.25 [−12.59, 2.10]

## Discussion

To our knowledge, the EASY model is the first intervention in middle-aged women to specifically target reduced sitting time as a catalyst for engaging in more physical activity. We demonstrated interest in our study and recruited an adequate number of participants to pilot the intervention. We also showed that we could deliver the EASY intervention as planned and the appropriateness of outcome measurements. Specifically, participants in both groups had a high level of satisfaction with the program, and we retained 92% of intervention group participants at 6 months. However, we note that for a larger trial, we will need to provide more time for recruitment and/or different strategies to meet recruitment goals. The study also supports the feasibility of using a novel activity monitor (the Fitbit) and online resources, to support women to be more active in their daily routine. The program was designed to instill confidence and knowledge about key elements of a sustainable physical activity program and to support people to make their own choices regarding an active lifestyle—rather than prescribing a specific one-size-fits-all program. One of the study participants expressed that the EASY model was “not just another walking program”—it was an opportunity for them to acquire skills and resources to manage their own physical activity.

In this study, sufficient participants were recruited in the timeframe to complete the pilot as planned. Although 30 participants declined participation, 21 stated it was because they were still working and unable to attend due to timing of the sessions. This was considered prior to the study; however, it was not possible to accommodate multiple sessions for the pilot study. The full protocol will provide sessions across a variety of days and times. Nonetheless, recruiting via local newspapers was successful here, and this mode and other recruitment techniques [57] will be employed for the future study. Another viable option for the next phase is to work with larger organizations to deliver the EASY model as a workplace intervention as part of a retirement package initiative, and in this way, it would be possible to utilize a known sampling frame to address wider generalizability of our findings.

Study participants had a high level of engagement with their attendance, and both groups rated their satisfaction with the program as 9/10. Reasons to explain this include living in a walkable city, the Fitbit monitor, financial incentives, and frequent contact. However, equally noticeable was the lower rate of retention for the control participants. Three participants dropped out before the study began because of group allocation; but notably, eight of the nine control participants completed the final assessment. The control participants who remained in the study had a high level of satisfaction, which may reflect group interactions with other study participants and/or program organization and delivery. Attrition rates for lifestyle interventions vary; in a Cochrane Review of lifestyle interventions for adults with diabetes, attrition rates ranged from 0–30% [58]. The results from the present study suggest that an RCT design for a larger trial, based on the current protocol, could introduce a potential loss to follow-up in the control group. Given the enthusiasm for the intervention and the readiness for change by study participants, a stepped wedge protocol [59] may enhance the study design.

Our secondary objectives were to assess the effect of the intervention on study participants’ physical activity and health outcomes. We note differences in step counts between groups at final assessment but observed that the groups were slightly different at baseline. This should be considered when interpreting the results and in the design of the future study (e.g., stratify participants by step counts above or below a cut point). An unanticipated finding in this study was a 4-kg weight loss, on average, favoring the intervention group, although we recognized that there were some differences in weight between groups at study commencement that may have had an effect on our results. Franz and colleagues [60] conducted a systematic review and meta-analyses of exercise-only interventions for weight loss. Minimal average weight loss at 12 months [1.9 (3.6) kg] in the exercise group was no better than advice alone. Thus, there is a clear role for dietary considerations in any study that aims to positively influence body weight. Although we provided one educational session on nutrition during a tour of a local grocery store with a dietitian and modelled healthy food choices with the lunches provided, dietary behaviors and body weight were not the focus of the study. Another possible explanation is that reductions in sitting time with increases in physical activity were “gateway behaviors” for changes in diet [61]. While Fleig and colleagues [62] noted that positive changes in physical activity occurred in parallel to nutritional changes, there are other interventions which did not note this synergy [63, 64]. Exploration of factors that contributed to change in body composition associated with our model would be an important focus for future studies.

Our study has limitations. First, the sample size was purposely small to assess key features such as participant recruitment and retention to guide the development and planning of the next phase of this research (e.g., interest in the study, feasibility of delivering the intervention, estimating a sample size for the larger study). Although our current strategies were successful in enrolling participants in a short time period, a recruitment strategy that included a known sampling frame (such as with a workplace intervention) would provide additional information to guide future studies. Second, the difference in outcomes between groups reported at 6 months are preliminary evidence that could be used to inform a larger trial, but these results may not be present in a scaled up version of the intervention. Third, the observed differences are likely conservative because of the lower number of control participants who completed the final assessment. In the larger trial, more sophisticated methods (including multiple imputations) will be used to address any missing data [65]. Finally, the EASY model adopts a multipronged approach; thus, it is difficult to discern the relative contribution of each element to any changes observed. Olander and colleagues [66] observed that effective BCTs for increasing self-efficacy in activity trials included action planning, time management, self-management of behavior, and social influences—components of which are contained within EASY. However, we observed a difference for the unadjusted measure of behavior intentions only but did *not* note a between group difference for self-efficacy measures. The area of determining which BCT was effective will be the focus of future studies that will be designed based on the findings herein.

## Conclusions

In conclusion, the EASY model was feasible to deliver in a community setting to women at retirement age. Participants were highly engaged in, and satisfied with, the EASY model, and 92% of intervention participants completed the final assessment at 6 months. It is promising that, despite our sample size, the intervention was an effective means to increase physical activity and decrease weight and blood pressure in this retirement age cohort. A phase III trial, using a different study design, is needed to ascertain the effectiveness of scaling up and long-term sustainability of the EASY model.

## Electronic supplementary material

Additional file 1: **Includes a participant handout and a summary of the EASY behavioral change techniques (BCTs).** Additional file 1: Figure S1 was a handout given to participants to review their current behavior and create a plan for their future activity patterns. Additional file 1: Table S1 explains in detail the BCTs employed with the EASY model. (DOCX 63 KB)
