# Supplementary material for: “Not just another walking program”: Everyday Activity Supports You (EASY) model—a randomized pilot study for a parallel randomized controlled trial
Source: Pilot Feasibility Stud. 2015 Jan 12;1:4. doi: 10.1186/2055-5784-1-4 (PMC4862805; doi:10.1186/2055-5784-1-4)
Supplement: Supplementary file 1 [file 40814_2014_3_MOESM1_ESM.docx]

**FIGURE S1.** This handout was given to participants to review their current behavior, and create a plan for their future activity patterns. An example was provided, with explanation on how to change, to highlight the gradual nature of the Everyday Activity Supports You (EASY) Model.

Movement Makeover

|  | Morning | Mid-morning | Lunch | Afternoon | Evening |
| --- | --- | --- | --- | --- | --- |
| BEFORE | Drive to work  Take elevator to office | Sit at desk while at work | Walk to store for a cup of tea | Drive home from work | Watch TV |
|  | | | | | |
| AFTER | Go for a walk before work; drive to office but get out 10 minutes early and walk the rest of the way to work | Take stairs while at office; using standing desk; set timer and get up every 30-40 minutes | Walk around the block; walk and talk meetings | Walk 10 minutes to location to meet drive | Short walk after dinner; water garden; only 1 hour TV time and get up 2-3 times; read a book |

How did I change?

January 2010

I recognized that I needed to change my sitting and physical activity patterns

Monitored my patterns with an activity monitor

Started by breaking up sitting time at work

Reduced my TV time at home; got up during commercials

Started to take short walks after meals

Added longer walks in the morning

**FIGURE S2.** This figure summarizes participants’ ideas generated through a three stage process: think about ideas related to the topic, share and discuss in a small group, and then come together in a larger group to compile the full list and categorize items into common themes.

Everyday Activity Supports You (EASY) Program Pilot

Brainstorm: Barriers to Activity and Coping Plans

| *Category* | *Reasons that prevent you from exercising* | *Coping Plans* |
| --- | --- | --- |
| **Health** | - Sick - Crisis, sick relative, etc. - Sore e.g., feet, knees, back, head, etc. - Feel stiff | - Don’t push yourself, and take a break if necessary - Take baby steps and build to your goals - Start doing things gradually to get active - Need to believe moving is beneficial to health - Buy decent and comfortable shoes - Remember your brain and memory can improve from exercise |
| **Motivation** | - Too tired - Not exciting - Procrastination - ‘Just don’t feel like it’ - Lack of incentives - My friends would laugh - Making up excuses - Plain laziness - ‘Walking is boring’ - ‘I’ll be the oldest and fattest one there’ - ‘I hate doing things I’m not good at and looking stupid’ | - Make a personal reward to completing the activity - Make a mantra, e.g., “I can do it!” - Remember the payoff – “you’ll feel good after you’re back home” - Create an opportunity to get out the front door - Make walks more social - Take new routes - Go to places of interest, ex. libraries, grocery stores, malls, etc. - Go to yoga classes if walking is boring - Listen to music or podcasts |
| **Safety** | - Safety | - Use common sense to stay safe! - Know the neighborhood, walk with a friend - Take identification on walks - Know where staircases exit |

| **Social** | - ‘I have no one to go with’ | - Walk with a dog - Meet up Group or Vancouver Walking Group - ‘No one left behind’ or ‘Fastwalk Group’ - There are coupons for activities (half price from online coupon groups). This could provide an opportunity to try new things (Dragon boating, kayaking, etc.) - Find someone to encourage you (husband, friend, etc.) |
| --- | --- | --- |
| **Time Management** | - ‘Feel like I have too much work to do’ - Other chores need to be done - Time, ‘Always rushing to appointments so I use the car’ - Personal Obligations | - Plan your day and include opportunities for being active - Try a planned walk with a different location - Organize clothes beforehand and put them out |
| **Weather** | - Too rainy/cold - Too hot - ‘It might rain’ | - Dress appropriately and take an umbrella - Walk at the mall, gym, or community centre if the sidewalks are too icy or slippery - Wear appropriate shoes - If hot, go for early or late walks - Shorten walks if hot - Bring water - Embrace the rain! - Buy rain gear that makes you happy (effective, fun, etc.), try thrift shopping |

**TABLE S1.** The table complements Figure 2 and provides an overview of the Behavior Change Techniques (BCT) (based on the BCT Taxonomy by Abraham and Michie [36,66]) that underpins the three main components of the Everyday Activity Supports You (EASY) Model.

| **EASY Model Component** | **Behavior Change Technique** | **Integration into Program** |
| --- | --- | --- |
| **Lunch & Learn Group Sessions** | | |
| *Monthly group sessions that provided practical information on ways to reduce sitting and increase daily physical activity. This component was 60 minutes in duration and occurred during the first hour of the 2-hour session.* | Action and Coping planning | *Think-Pair-Share* format. Participants were asked to brainstorm ideas around action and coping planning. They discussed brainstorming ideas in smaller groups, wrote these ideas on sticky notes and then presented them to the larger group. The larger group sorted the ideas by concepts. |
|  | Barrier identification | *Think-Pair-Share* format. Participants were asked to brainstorm ideas around barrier identification. They discussed brainstorming ideas in smaller groups, wrote these ideas on sticky notes and then presented them to the larger group. The larger group sorted the ideas by concepts (see example from one of the sessions) |
|  | Behavior substitution | Discussion on ways to change behavior (see Movement Makeover handout) |
|  | Goal setting | Presentation on why and how to set goals |
|  | Habit reversal | Discussion on ways to change behavior |
|  | Shaping knowledge | Monthly educational topics via formal presentation and small group discussion |
|  | Social support | Group discussion and brainstorming opportunities |
| **Activity 4-1-1 Individual Sessions** | | |
| *During the second hour, participants engaged in two different elements: a 10 minute one-on-one individual session with an exercise professional (called Activity 4-1-1) and a concurrent group session on brainstorming ideas for self-management as described above.* | Action and Coping planning | Participants individually discussed with the exercise professionals their plans and strategies to cope with setbacks. |
|  | Barrier identification and problem solving | Participants individually discussed with the exercise professionals their barriers and strategies to cope with setbacks. |
|  | Behavior substitution | Exercise professionals reviewed strategies, and made suggestions to reduce sitting and increase physical activity. |
|  | Graded tasks | The EASY model is a very gradual increase in activity. Participants also recorded their daily stepcount and reviewed their progress with the exercise professionals. At each session, participants were encouraged to only increased stepcount by 5%. |
|  | Goal setting | Participants set their activity goals with the exercise professionals |
|  | Habit reversal | Exercise professionals reviewed strategies with participants during session. |
|  | Review Goals | Participants individually reviewed goals with exercise professionals at each session. |
| **Self-Directed Activities** | | |
| *Participants were provided with a Fitbit activity monitor and bus tickets to encourage adoption of more physical activity, and specifically active transportation.* | Prompts/cues  (bus tickets) | Participants were provided with two sets of 10 bus tickets to encourage use of public transportation. They were also given a 1-hour session from the local public transportation company on how to use the system. |
|  | Self-monitoring of behavior  (Fitbit) | Participants were provided with an activity monitor, and group and individual instructions on how to use it, the online program and/or smartphone application. |
